# Impact of High-Carbohydrate Diet on Metabolic Parameters in Patients with Type 2 Diabetes

**DOI:** 10.3390/nu9040322

**Published:** 2017-03-24

**Authors:** Chan-Hee Jung, Kyung Mook Choi

**Affiliations:** 1 Division of Endocrinology and Metabolism, Department of Internal Medicine, Soonchunhyang University School of Medicine, Bucheon Hospital, 170 Jomaru-Ro, Wonmi-Gu, Bucheon-Si, Gyeonggi-Do 420-767, Korea; chanh@schmc.ac.kr; 2 Division of Endocrinology and Metabolism, Department of Internal Medicine, Korea University Guro Hospital, 80 Guro-Dong, Guro-Gu, Seoul 152-050, Korea

**Keywords:** high carbohydrate, type 2 diabetes, fiber, glycemic index, glycemic load

## Abstract

In patients with type 2 diabetes mellitus (T2DM), whether dietary carbohydrates have beneficial or detrimental effects on cardiometabolic risk factors has drawn attention. Although a high-carbohydrate (HC) diet and a low-carbohydrate (LC) diet have gained popularity for several decades, there is scarce review focusing on the effects of HC diet on glucose, lipids and body weight in patients with T2DM. In this review, we examined recently-published literature on the effects of HC diets on metabolic parameters in T2DM. HC diets are at least as effective as LC diets, leading to significant weight loss and a reduction in plasma glucose, HbA1c and low density lipoprotein-cholesterol (LDL-C) levels. The major concern is that HC diets may raise serum triglyceride levels and reduce high density lipoprotein-cholesterol (HDL-C) levels, increasing the risk of cardiovascular disease. However, these untoward effects were not a persistent consequence and may be ameliorated with the consumption of a low glycemic index (GI)/low glycemic load (GL) and high fiber. Carbohydrate intake should be individualized, and low caloric intake remains a crucial factor to improve insulin sensitivity and reduce body weight; however, an HC diet, rich in fiber and with a low GI/GL, may be recommendable in patients with T2DM.

## 1. Introduction

Dietary recommendations for carbohydrates in T2DM have changed over the past decades. The proper amounts and types of carbohydrates have been an area of persistent controversy in the management of T2DM. Before the availability of insulin, a low-calorie diet, based on a strict restriction of carbohydrate intake and an increased intake of dietary fat, was recommended [1,2]. The discovery of insulin allowed for a higher carbohydrate diet. Furthermore, as the importance of cardiovascular complications in diabetes became recognized, a high-carbohydrate, low-fat (HCLF) diet was recommended, for the prevention cardiovascular disease (CVD) in patients with T2DM. The rationale for the a high-carbohydrate (HC) diet was that this diet did not increase fasting plasma glucose (FPG) and reduced serum cholesterol, which was beneficial in the context of CVD [3,4,5,6,7]. However, despite this trend, the prevalence of metabolic diseases, such as obesity and T2DM, have increased, and untoward effects of HC diet, such as postprandial glucose elevation, hyperinsulinemia and lipid concern (increasing triglycerides and decreasing HDL-C), also made a reappraisal of a low-carbohydrate (LC) diet. The American Diabetes Association (ADA) recommends monitoring carbohydrate intake to achieve glycemic control [7]. This recommendation is based on studies showing reduced postprandial glucose and triglyceride responses in subjects consuming lower carbohydrates (40% vs. 60% carbohydrate) [8,9]. A low-carbohydrate, high-fat (LCHF) diet has attracted attention as a means of weight reduction and blood glucose control in T2DM. However, despite the short-term advantage, an argument has arisen due to the lack of evidence related to long-term benefit and safety.

One of the most significant changes in medical nutrition therapy (MNT) for diabetes is the concept of individualized macronutrient distribution, rather than the prescription of a standardized proportion of macronutrients. However, there is accumulating evidence that changes in diet macronutrient composition facilitate weight loss, improving glucose control and lipid profiles in patients with T2DM. Generally, the amount of carbohydrate ingested is regarded as the biggest factor of postprandial glucose and insulin response and dissimilar metabolic parameters; however, the quality of carbohydrate has also come under scrutiny.

Moreover, although the effects of LC diets have been described extensively in focused reviews and clinical studies, there is scarce review focusing on the effects of HC diets over recent years. Based on the above considerations, this review will examine currently available publications on the metabolic effects of HC diets, including the impact of its quantity and quality, in patients with T2DM.

## 2. Obstacles in Evaluating High-Carbohydrate Diet Studies in Diabetes Care

The lack of a standardized definition for the term “high-carbohydrate” is the greatest obstacle to interpret the findings of the studies. The HC diet is a rather relative concept, for example a 50% carbohydrate composition from total energy can be labeled as a ‘higher carbohydrate diet’ if the diet of the comparison group is <50% carbohydrate or a ‘lower carbohydrate diet’ if the diet of the comparison group is a >50% carbohydrate diet. In addition, altering the level of carbohydrate affects the proportion of other macronutrient (fat and protein), making it more difficult to isolate the pure effect of carbohydrate proportion.

### 2.1. Definition of High-Carbohydrate Diet

There is no universal definition amongst researchers regarding the amount of carbohydrate in HC diets, and studies apply different standards (Table 1) [10,11,12,13,14]. According to the authors, carbohydrate amount was presented as % of total energy intake or gram/day. As shown in Table 1, the definition of HC or LC can be relative; therefore, it would seem to be more appropriate to use the term higher or lower carbohydrate diet.

In a meta-analysis, Kodama et al. used the carbohydrate/fat ratio (C/F ratio), instead of the % of total energy [14]. They assigned one diet as the HCLF diet, which was defined as having a relatively high C/F ratio, and the other as the LCHF diet, which had a relatively low C/F ratio. They suggested that the C/F ratio ranged from 0.60–1.56 for the LCHF diets and from 1.67–7.30 for the HCLF diets. The conflicting definition of HC or LC diet may be partly due to the lack of an optimal range of carbohydrate intake.

In this review, we selected articles to review not by the publishing author’s definition of HC, but by following our own standard: any carbohydrate intake less than 45% of total energy as equal to very low carbohydrate (VLC); 45%–49% as equal to low carbohydrate (LC); 50%–54% as equal to moderate carbohydrate; 55% or more as equal to high carbohydrate (HC).

### 2.2. Optimal Range of Carbohydrate

A range of cut-off values for the optimal carbohydrate intake has been recommended by various authorities (Table 2) [15,16,17,18,19,20]. Recent guidelines recommend individualized macronutrient distribution based on the eating patterns, preferences, cultural factors and metabolic goals of each patient with T2DM and emphasize the quality of macronutrients. Although the ideal amount of carbohydrate is undecided, various guidelines recommend that the percentage of total daily energy from carbohydrate should be no less than 45% (especially, because the staple food of many Asian populations, such as Korea, China and Japan, is rice based on carbohydrate, the standard level is higher, such as 50%) to prevent high intakes of fat. In addition, carbohydrate may contribute to up to 60% of total energy if the carbohydrate source can be derived from a low glycemic index (GI) and high fiber.

## 3. Literature about the Effects of High Carbohydrate on Metabolic Parameters in Type 2 Diabetes

We conducted electronic searches of the following databases: PubMed, Medline, OVID Medicine, EMBASE, Web of Science, Cochrane Library, KoreaMed, MEDRIC and KSI KISS, including all studies published from 2010 to the present. A combination of exploded medical subject headings (MeSH) and free text searching were used, including ‘Type 2 diabetes’, ‘NIDDM’, ‘high carbohydrate’, ‘higher carbohydrate’, ‘carbohydrate rich’, ‘high glycemic index’, ‘low glycemic index’, ‘glycemic load’, ‘hemoglobin A, glycosylated’, ‘fasting plasma glucose’, ‘postprandial glucose’, ‘insulin sensitivity’, ‘insulin resistance’, ‘cholesterol’, ‘triglyceride’, ‘HDL-cholesterol’, ‘LDL-cholesterol’, ‘body weight’ and ‘obesity’. The search was limited to studies written in the English language and adults above 19 years.

### 3.1. Studies on the Effects of High-Carbohydrate Diet or High-Carbohydrate Diet vs. Low-Carbohydrate Diet

A retrospective study was conducted to document the effects of eating an HCLF (Carbohydrate(C):Fat(F):Protein(P) = 81%:7%:12%), moderate-sodium and purely plant-based ad libitum diet for seven days on the biomarkers of CVD and T2DM from 1615 participants in Santa Rosa, California [21]. The majority of patients were female (65.1%), white non-Hispanic (92.1%), Asian (2%) and with a median (interquartile range: IQR) age of 58 (18) years. Among participants, patients with DM were 14.4%. Meals were based around common starches with the addition of fresh fruits and non-starch green, orange and yellow vegetables. No animal-derived ingredients and no isolated vegetable oils were used. Even though most antihyperglycemic medications were reduced or discontinued at baseline, blood glucose by a median (IQR) of 3 (11) mg/dL was decreased (*p* < 0.001). The median (IQR) weight loss was 1.4 (1.8) kg (*p* < 0.001), and the median (IQR) decrease in total cholesterol was 22 (29) mg/dL (*p* < 0.001). This study showed that an HC, high fiber diet for only seven days allows overweight patients to lose weight and improve metabolic profiles very early even if they ate ad libitum. Guldbrand H et al. conducted a prospective randomized parallel trial involving 61 Swedish adults with T2DM comparing the effects of a two-year intervention with an HC diet or an LC diet [22]. Patients were randomized to either an HC diet (C:F:P = 55%–60%:30%:10%~15% of total energy) or an LC diet (C:F:P = 20%:50%:30% of total energy), each with an energy content of 1600 kcal/day for women or 1800 kcal/day for men. The HC diet had a nutrient composition that was similar to that traditionally recommended for the treatment of T2DM in Sweden. The largest changes in macronutrient intake were seen in patients randomized to the LC group. Indeed, patients in the HC group had the same macronutrient composition at baseline during the study. Furthermore, they reported about taking DM medication, including insulin, metformin and sulfonylurea and no difference between the two diets at the baseline and during the study period. There was no difference in weight reduction between the groups (at six months, HC diet group, −4.0 ± 4.1 kg; LC diet group, −4.3 ± 3.6 kg; and at two years, HC diet group, −2.97 ± 4.9 kg; LC diet group, −2.34 ± 5.1 kg; *p* = 0.33 for all time points between groups). However, a reduction in HbA1c was observed in the LC diet group only at six months (−4.8 ± 8.3 mmol/mol, *p* = 0.004). The reduction in HbA1c level was statistically significant within the LC group (*p* = 0.005 for all time points), but did not differ between the groups when compared at all time points (*p* = 0.76). While insulin doses were reduced significantly more with the LC diet group at six months (at baseline, LC diet group 42 ± 65 unit, HC diet group 39 ± 51 unit; six months, LC diet group 30 ± 47 unit, HC diet group 38 ± 48 unit; *p* = 0.046 for between-group change). Regarding lipid profile, at six months, the HDL-C level increased in the LC diet (from 1.13 mmol/L–1.25 mmol/L, *p* = 0.018), while LDL-C did not differ between the two groups.

In addition, Brehm et al. compared the effects of MUFA diets (C:F:P = 45%:40% (with 20% MUFA):15%) and HC diets (C:F:P = 60%:25%:15%) on body weight and glycemic control in 124 overweight/obese patients with T2DM (92 Caucasians and 32 African Americans) for one year [23]. Compared with the HC diet, the high MUFA diet included fewer servings of starches, fruit and meat and more servings of fat (olive and canola oils). Compared with baseline participants in both groups, the increased intake of vegetables and fruits (*p* < 0.001) was reported, with the HC group consuming even more fruit than the high MUFA group at 12 months (*p* = 0.012). This study showed that both diets showed similar weight loss (−4.0 ± 0.8 kg vs. −3.8 ± 0.6 kg, *p* = 0.720) and comparable beneficial effects on HbA1c, FPG, insulin and HDL-C. The overall retention rate was 77%, with 69% for the high MUFA diet group and 84% for the HC diet group (*p* = 0.06). Only three participants left the study because of diet-related reasons. A follow-up assessment of a subset of participants was conducted 18 months and these participants maintained their weight loss and HbA1c. Furthermore, the high MUFA diet presented a greater change in typical patterns of macronutrient intake than the HC diet, and the extension study suggested a shift back to more usual food choices after the intervention was completed. One study evaluated the lipid profile and inflammatory markers in 48 Indians with T2DM after fasting, 2 h and 4 h after a mixed meal with HC (C:F:P = 79.1%:13.2%:7.7%) [24]. Consumption of an HC meal was associated with a rise in triglyceride (39.9 mg/dL increase at 2 h and 109 mg/dL at 4 h) and fall in total cholesterol (6.5 mg/dL decline at 2 h and 2.7 mg/dL at 4 h), HDL-C (2.7 mg/dL decline at 2 h and 2.2 mg/dL after 4 h) and LDL-C (6.5 mg/dL decline at 2 h and 5 mg/dL at 4 h), in subjects with T2DM.

A meta-analysis by van Wyk HJ et al. reviewed 12 RCT examining the effects of an HC diet compared to an LC diet in T2DM over a minimum period of four weeks [25]. The authors concluded there were no significant differences in metabolic markers, including glycemic control, between the two diets, although one study found that weight loss on an LC diet was greater [26]. In summary, there appear to be no differences between an HC diet and an LC diet in terms of metabolic markers and glycemic control.

### 3.2. Studies on High-Carbohydrate Diet vs. High-Protein, Low-Carbohydrate Diet

An RCT compared HC diets and high-protein (HP) diets over two years in 419 overweight patients with T2DM (The Diabetes Excess Weight Loss; DEWL Trial) in New Zealand [27]. Patients were prescribed either an HC (C:F:P = 55%:30%:15%) or HP-VLC (C:F:P = 40%:30%:30%) diet for one year and were further assessed 12 months after intervention. The aim of both interventions was to reduce total energy intake approximately 500 kcal/day. The study was completed by 70% (294/419). Weight loss (2–3 kg, *p* < 0.001) occurred in both groups by 12 months and was largely maintained over the following 12 months. No differences between groups were found in body weight changes during the intervention phase or the 12-month follow-up (*p* = 0.8). There was no significant difference at any time point or overall between the groups in HbA1c (*p* = 0.9). In addition, no significant differences between groups were found in lipid profiles in the final results at 24 months. Regarding triglycerides, triglyceride levels decreased more in the HP-VLC group between baseline and 12 months, but both groups had returned to baseline levels by 24 months. Larsen et al. conducted a 12-month RCT to investigate the effect of HC (C:F:P = 55%:30%:15%, *n* = 46) or HP-VLC diet (C:F:P = 40%:30%:30%, *n* = 53) in 99 patients with T2DM in Australia [28]. The study consisted of two dietary periods: a three-month 30% energy restriction period followed by nine months of energy balance, and both diet groups were recommended a low GI diet. Eighteen-point-nine percent of the HP-VLC group and 19.6% of the HC group discontinued the diet. The proportion of individuals who were lost to follow-up was slightly higher in the HP-VLC group (9.2% vs. 4.2%). As a primary outcome, HbA1c decreased in both groups over time, with no significant difference between groups (mean difference of the change at 12 months; 0.04 (−0.37, 0.46); *p* = 0.44). Both groups also demonstrated decreases over time in weight, serum triglyceride and total cholesterol and increases in HDL-C. These parameters were no different between groups (mean difference of the change at 12 months; −0.07 (−1.67–1.54), −0.17 (−0.65–0.32), −0.16 (−0.51–0.18), 0.01 (−0.10–0.11)). Therefore, two studies suggested that there is no superior long-term metabolic benefit of an HP diet over an HC in the management of T2DM.

### 3.3. Studies on High-Carbohydrate Diet in Asians

A Western dietary pattern is typically high in fat and animal protein, whereas plant-based carbohydrates, for example rice, constitute a significant part of the Asian diet. Thus, carbohydrate intake takes up a relatively high proportion of macronutrients in Asian patients with T2DM. In a Korean study, the distribution of macronutrients (C:F:P, % of total energy) was reported as 68%:16%:16%, respectively [29]. According to data from eastern China, in patients with T2DM, the proportion of calories derived from carbohydrate, fat and protein were 61.4%, 25.4% and 13.3%, respectively [30]. As we can see from the Asian data, an HCLF pattern (C:F:P = 60%:16%–25%:15%–24%) is predominant in Asian patients with T2DM. However, studies on HC in Asian are relatively sparse.

Hsu et al. explored the metabolic responses in 28 East Asian Americans (Korean, Japanese and Chinese) and 22 Caucasian Americans at risk of developing T2DM when transitioning from a traditional Asian diet (C:F:P = 55%~70%:15%:15%, 33 g/day fiber) to a typical Western diet (C:F:P = 50%:34%:16%, 10–12 g/day fiber) for six weeks [31]. All participants had either a family history of T2DM and/or a history of gestational DM, impaired fasting glucose or impaired glucose tolerance. While on traditional Asian diet (HCLF), improvements in insulin AUC (decreased insulin AUC; −960.2 µU/m L × h, *p* = 0.001) and decreases in body weight (−1.6 kg; *p* < 0.001) and body fat (−1.7%, *p* < 0.001) were observed in both East Asian American and Caucasian Americans. Despite efforts to maintain an iso-energy state and consumption of similar energy, traditional Asian diet (HCLF) induced weight loss and improved insulin sensitivity in both East Asian Americans and Caucasian Americans. Total cholesterol (*p* < 0.001), HDL-C (*p* < 0.001) and LDL-C (*p* < 0.001) were significantly decreased during the traditional Asian diet while increased during the traditional Western diet. These beneficial effects on weight loss, glucose, insulin sensitivity and lipid profile are likely to be due to the high fiber content of the traditional Asian diet (HCLF).

Kamada C et al. performed a study to assess the correlation between the C:F:P energy ratio and HbA1c and TG in 1173 Japanese elderly patients with T2DM, aged 65 years or older [32]. They divided participants into four groups by the % of total energy intake of carbohydrate (C1: <55%; C2: 55%≤, <60%; C3: 60≤, <65%; C4: ≥65%). They reported that the carbohydrate energy ratio has no correlation with HbA1c levels. However, in patients with 65% or more of carbohydrate intake, serum triglyceride levels exceeded 150 mg/dl. Therefore, the recommended proportion of carbohydrate energy for elderly Japanese with T2DM was suggested to be less than 65%.

In a study examining nutritional intake in good and poor glycemic control groups in 48 elderly Korean patients with T2DM, the proportions of macronutrients differed significantly between the good glycemic group (C:F:P = 62%:22%:19%) and the poor glycemic control group (C:F:P = 71%:15%:15%) [33]. The macronutrient distribution in the good glycemic control group was comparable to the distribution recommended by the Korean Diabetes Association (KDA) (C:F:P = 60%:20%:20%). Lower intakes of protein (*r* = −0.338, *p* < 0.05) or fat (*r* = −0.385, *p* < 0.01) were related to higher HbA1c levels, while there was no association between carbohydrate intake and HbA1c levels observed in the study.

Indeed, studies performed in Western populations tend to set lower carbohydrate amounts as a definition of HC diet. Several studies considered it to be an HC diet if carbohydrate intake is over just 50% of total energy intake [34,35]. We guessed them as moderate carbohydrate diets (50%–54%), which were defined as HC diets in their studies, so we did not include them in this review [34,35]. On the other hand, the above-mentioned studies which were conducted in Asians generally set the standard of an HC diet as a carbohydrate intake over 60% of total energy.

### 3.4. Adherence and Sustainability of HC Diet

Adherence to dietary regimens is an important factor to obtain and maintain any diet-induced clinical improvements. Adherence rate of currently-published RCT of HC diets was 65%–84%, and this was not inferior to the LC diet adherence rate [22,23,26]. Moreover, studies of HC diet with high fiber showed good adherence of participants [36,37].

However, long-term patient adherence to and sustainability of the recommended diet are often difficult to achieve. The above-mentioned DEWL Trial showed that in both HPVLC and HC diet, the macronutrient composition trended back to baseline proportions between six months and two years, emphasizing the difficulty in making sustained changes to these [27]. There is a study that shows the difficulty to sustain moderately-restricted carbohydrate. A total of 144 obese, T2DM participants were randomly assigned to an LC diet (<30 g/day, encouraged to consume healthy fats) or to an LF diet (≤30% of calories from fat, <7% of total calories from saturated fats, <300 mg of dietary cholesterol, with a deficit of 500 kcal/day) [38]. At Month 6, the LC group had a clinically significant reduction in HbA1c of −0.5% (compared to −0.1% in the low-fat condition), although this was not sustained over time. By Month 24, mean HbA1c levels decreased from baseline by 0.1% and 0.2% in each group, respectively. Lipid profiles and dietary intake did not differ between the groups at Month 24 (or at Months 6 or 12). Baseline carbohydrate intake in the LF group was 43.2%, and it increased by only 3.5% at Month 24. In this study, an LF diet did not mean HC diet, and meaningful higher carbohydrate consumption was not achieved finally (regarding carbohydrate intake; increased by 3.5% from 43.2% of baseline). In the LC group, there was a modest reduction in carbohydrate intake at six months, which gradually increased over the next 18 months. Both groups failed to achieve their dietary targets throughout the duration of the study. Furthermore, participants in the two groups appeared to consume similar diets, despite the prescription of markedly different dietary intake. This suggests that carbohydrate-restricted diets may be hard to sustain. In addition, a 24-month study based on a low-intensity intervention, approximating what is deemed feasible in outpatient practice, showed how sustainability is difficult to maintain [27,38].

### 3.5. Various Circumstances Influence the Response to a High-Carbohydrate Diet

Other dietary components, fat or protein may also influence the response to an HC diet or be inextricably linked to this. Allerton et al. assessed postprandial metabolic and appetite responses of co-ingestion of whey protein with an HC breakfast (C:F:P = 74%:3%:22%) compared with consumption of HC only (C:F:P = 87%:4%:8%) [39]. Adding whey protein to an HC breakfast was found to enhance the acute postprandial insulin response (*p* < 0.05), without influencing glycemia (*p* = 0.247) or appetite responses (*p* > 0.05), following a subsequent mixed-macronutrient meal. In addition, other macronutrient manipulation can affect HC on glycemic and insulinemic response. For example, the co-ingestion of 68 mL of amino acid mixture with white rice (carbohydrate-rich food with a high GI and high glycemic response) reduced the peak blood glucose (*p* < 0.05) and glycemic response of white rice (*p* < 0.05), without increasing the insulinemic response [40].

Significant gene-nutrient interactions can also affect the response to HC. A study by Ramos-Lopez et al. reported that a specific genotype was associated with higher carbohydrate intake [41]. Researchers found that the sweet taste receptor TAS1R2 polymorphism (Val191Val) was associated with a higher carbohydrate intake (% of total energy intake: 58.5% vs. 52.4% vs. 53.1%, *p* = 0.04) and hypertriglyceridemia (194 vs. 169 vs. 150 mg/dL, *p* = 0.02) than other genotype carriers (Ile/Ile or Ile/Val) among the population of West Mexico. Another study reported that carbohydrate intake interacts with the SNP276G>T polymorphism in the adiponectin gene to affect FPG, HbA1c and HDL-C in Korean patients with T2DM [42]. The levels of carbohydrate intake were categorized as <55%, 55%–65% and >65% of total energy intake. The G allele was associated with higher FPG only in subjects consuming a lower carbohydrate diet (<55% of energy). However, when carbohydrate intake was intermediate (55%–65%), carriers of the T allele had greater FPG and HbA1c concentrations. When carbohydrate intake was high (>65%), carriers of the T allele had greater HDL-C concentrations. Furthermore, evidence of genetic variation associated with greater improvement of glucose homeostasis in individuals who choose an HCLF and high-fiber diet have been reported [43,44]. In a study by Qi et al., the T allele of the glucose-dependent insulinotropic polypeptide receptor (GIPR) rs2287019 genotype was marginally associated with greater weight loss (*p* = 0.06) in obese adult participants who were assigned to HC diets (C:F:P = 65%:20%:15% or 55%:20%:25%) and with greater decreases in FPG (*p* = 0.006), fasting insulin (*p* = 0.03) and HOMA-IR (*p* = 0.01). In the LC or VLC diet groups (C:F:P = 45%:40%:15% or 35%:40%:25%), there was no significant genotype effect on changes (all *p* > 0.44) [43].

## 4. Are All Carbohydrates the Same? Does Carbohydrate Type Matter?

There is increasing evidence that carbohydrate type, in addition to total carbohydrate amount, is also important [45]. Different blood glucose responses have been demonstrated even after consumption of identical amounts of carbohydrate [45,46]. Various carbohydrate subtypes have specific structures and properties that, either alone or in combination with other nutrients, are likely to influence appetite, blood glucose responses and energy balance [47]. An HC diet with substantial amounts of sugars or refined carbohydrates and low in fiber may cause a transient increase in triglycerides and a decrease in HDL-C in some individuals [48,49]. The high sugar diet appeared to stimulate food intake, while complex carbohydrates are likely to inhibit the food intake effect.

### 4.1. Glycemic Index/Glycemic Load

There has been concern for a long time about some possible unfavorable effects of carbohydrate on postprandial glycemic and insulin response and plasma lipid levels, an increase of triglyceride and a decrease of HDL-C level. However, it has been thought that these untoward effects on glucose and some lipid profiles are largely due to a temporary and rapid perturbation induced by HC diet. Therefore, it has been thought that these could be ameliorated if carbohydrate digestion and absorption slacken off.

The glycemic index (GI) measures the ability of a carbohydrate food to raise the blood glucose level [50]. Low-GI diets have shown beneficial effects on glucose control in relatively short-term trials in patients with T2DM, although the long-term role and utility of the low-GI remains unclear [51,52,53]. However, the OmniCarb RCT comparing high-GI (≥65% on the glucose scale) and low-GI (≤40% on the glucose scale) diet based on a healthful Dietary Approaches to Stop Hypertension (DASH)-type diet did not find any differences in insulin sensitivity, systolic blood pressure or lipid profiles after five weeks of controlled feeding [54].

A meta-analysis of 14 RCT (RCT for T1DM, 8 RCT for T2DM and 1 RCT for both T1DM and T2DM, duration range 12 days–12 months, mean 10 weeks) determined whether low-GI diets (average GI = 65), compared with high-GI diets (average GI = 83), improved overall glycemic control [55]. This meta-analysis showed that low-GI diets reduced HbA1c by 0.43% [0.13–0.73] more than high-GI diets in individuals with DM. An RCT of 121 patients with T2DM compared patients on a high wheat fiber diet (C:F:P = 48.3%:28.5%:21.4%, 18.5 g/1000 kcal fiber, GI = 82) and patients on a low-GI legume diet (C:F:P = 45.4%:30.5%:22.8%, 25.6 g/1000 kcal fiber, GI = 66) over three months [56]. The mean HbA1c fell by −0.5% absolute HbA1c value (95% CI, −0.6%–−0.4%) on the low-GI legume diet and by −0.3% absolute HbA1c value (95% CI, −0.4%–−0.2%) on the high wheat fiber diet. The relative reduction in HbA1c levels after the low-GI legume diet was greater than that after the high wheat fiber diet by −0.2% (*p* < 0.001). Regarding the lipid profile, the low-GI legume diet produced a significant decrease in total cholesterol level (−8 mg/dL, *p* < 0.001) and triglyceride levels (−22 mg/dL, *p* < 0.001) with no significant change in HDL-C level (−1 mg/dL, P=0.19). The relative reduction in total cholesterol (−9 mg/dL vs. −2 mg/dL, *p* = 0.005) and triglyceride (−21 mg/dL vs. −9 mg/dL, *p* = 0.03) was greater in the low-GI legume diet than the high wheat fiber diet. Another meta-analysis by Thomas and Elliott was reported after subtracting two RCT of less than a four-week duration and adding new RCT published after publication of the meta-analysis by Brand-Miller [57]. This analysis also showed a significant decrease in HbA1c (-0.43%, (−0.7–−0.2), *p* < 0.001) with a low-GI diet than with a high-GI diet. In the Canadian Trial of Carbohydrates in Diabetes (CCD), the authors compared the effects of LC/high-GI (C:F:P = 47%:31%:22%, GI = 63), moderate carbohydrate/low-GI (C:F:P = 52%:27%:21%, GI = 55) or VLC/high MUFA (C:F:P = 39%:40%:21%, GI = 59) diets on HbA1c, plasma glucose, lipids and CRP for one year in patients with T2DM with optimal glucose control treated by diet only [52]. There were no differences in long-term HbA1c between the diets. After 1 y of the low-GI diet, 2-h post-load glucose levels were ~1 mmol/L lower than those seen with the other two diets (*p* = 0.001). With the low-GI diet, mean triglyceride was 12% higher and HDL-C was 4% lower than that with the VLC diet (*p* < 0.05), but the difference in the ratio of total cholesterol:HDL-C disappeared by six months (time × diet interaction, *p* = 0.044).

Argiana et al. reported that consumption of desserts with a low GI to glycemic load (GL) ratio in a balanced hypo-caloric diet had a positive impact on anthropometric and glucose control in patients with T2DM [58].

Low-GI foods are generally rich in fiber, so there is limited evidence that a low-GI diet can improve long-term blood glucose control in subjects with T2DM, independently of its fiber content [59]. Therefore, GI values should not be used in isolation, but should be interpreted in relation to other relevant food characteristics, e.g., fiber content, energy density, content of other macronutrients and available carbohydrates. Moreover, a one-year controlled trial of the effects of a low-GI on HbA1c has been reported; however, studies with a longer follow-up period are required to determine the feasibility of incorporating a low-GI diet as part of a lifestyle and to assess long-term glycemic control [52].

An important determinant of glycemic response to a consumed carbohydrate is not only GI, but also the total amount of carbohydrates ingested. The GL is the product of GI and the amount of carbohydrate in the food (GL = GI × available carbohydrate (g)/100) [60]. In epidemiologic studies, the concept of GL was developed to better represent both the quantity and quality of the carbohydrate consumed [61]. Some previous studies have examined regarding the different impact of GI and GL on glucose control. A cross-sectional analysis of RCT study of 227 obese Japanese (the Saku Control Obesity Program) investigated the relation between the GI or GL and glycemia and other metabolic risk factors. The average dietary GI was 66 ± 5, and the average dietary GL was 79 ± 17 (1000 kcal). They showed that GI was not associated with HbA1c, but GL was positively associated with HbA1c [62]. For increasing quartiles of GI, the adjusted mean HbA1c were 6.3%, 6.7%, 6.4% and 6.4% (*p* for trend = 0.991); whereas, for increasing quartiles of GL, the adjusted mean HbA1c were 6.2%, 6.2%, 6.6% and 6.5% (*p* for trend = 0.044). The adjusted odds ratio (95% CI) for HbA1c  ≥ 7.0% among participants with higher GL (≥median) was 3.1 (1.2–8.1) compared to the participants with a lower GL (< median). Further, among participants with FPG  ≥ 150 mg/dL, 81.3% had a higher GL; the adjusted odds ratio (95% CI) for FPG  ≥ 150 mg/dL among participants with a higher GL was 8.5 (1.7–43.4) compared to those with a lower GL. Conversely, GI and GL were not associated with lipid levels and adiposity measures other than glycemia. Another cross-sectional study of 640 T2DM patients aged 28–75 years revealed that the odds ratios of the highest quartile of GL (>212.0) compared with the lowest quartile of GL (<129.6) were 2.58 (1.08–6.15) for elevated FPG (>130 mg/dL) (*p* trend = 0.02) and 3.05 (1.33–7.03) for elevated HbA1c (>8.6%) (*p* trend = 0.008) [63]. After additional adjustment for dietary fiber, the relation of GL with elevated FPG and HbA1c was significantly sustained (OR 3.0 (1.22–7.33), *p* for trend = 0.007, OR 3.94 (1.66–9.31), *p* for trend = 0.002, respectively). However, GI was not significantly associated with either elevated FPG or HbA1c. The odds ratios of the highest quartile of GI (>63.5) compared with lowest quartile of GI (<54.1) were 1.41 (0.84–2.38) for elevated FPG (*p* for trend = 0.19) and 1.42 (0.86–2.35) for elevated HbA1c (*p* for trend = 0.2). The previous ADA statement mentioned that the use of GI and GL may provide a modest additional benefit for glycemic control over that observed when total carbohydrate is considered alone. However, the current ADA focused GL only instead on the use of the both GI and GL for glucose control [15]. However, the limitations of the GI and GL need to be taken into account because there are various factors causing controversies that enclose the results of GI versus GL of foods, such as the property of food, inter-and intra-individual variability, unstandardized methods for test of GI and health status, race and gender of study subjects for defining GI [64,65].

### 4.2. Dietary Fiber

Dietary fiber is defined as carbohydrate polymers with more than a three degree polymerization, which are neither digested nor absorbed in the small intestine [66]. Furthermore, they require bacterial fermentation located in the large intestine as a group of substances in plant foods that cannot be completely broken down by human digestive enzymes [67]. Dietary fiber can be divided into many different types. Among types, non-starch polysaccharides and oligosaccharides can be divided into groups of soluble and insoluble [68]. Many of the beneficial effects of dietary fiber to the management of T2DM may be derived either by soluble or insoluble dietary fiber [65,69,70]. Especially soluble viscous dietary fiber tends to increase the viscosity or thickness of the intestinal contents, which delays gastric emptying, decreasing the absorption of macronutrients, resulting in lower postprandial glucose responses and reducing total and LDL-C through the increase of the rate of bile excretion [71,72]. Insoluble dietary fiber increases the passage rate of nutrients through the GI tract, thus resulting in a decreased absorption of carbohydrates and promotes insulin sensitivity and reducing postprandial glucose response through short chain fatty acids via fermentation [73,74]. In addition, fiber is processed more slowly, promoting earlier satiety and weight loss [75,76]. Forty years ago, a clinical study showing the remarkable benefit of HC, high fiber diet on glucose control was reported [77]. Thirteen hyperglycemic men with DM were fed HC diets rich in fiber (C:F:P = 75%:9%:16% with crude dietary fiber averaged 14.2 g) for two weeks. Of these, five were treated with 15–28 units of insulin/day, 5 were treated with sulfonylureas and 3 were treated with 40–55 units of insulin/day. All subjects were fed ADA diets (C:F:P = 43%:34%:23% with crude dietary fiber averaged 4.7 g) for one week, followed by an HC diet rich in fiber for two weeks. After two weeks on a 75% HC diet rich in fiber, FPG levels were significantly lower (*p* < 0.001). Sulfonylureas were discontinued in all five men, and insulin was discontinued in four men. Besides, fasting serum total cholesterol levels were significantly lower (*p* < 0.001), and fasting triglyceride levels were lower on the HC diet rich in fiber than on the ADA diet (*p* < 0.05).

A randomized, controlled, open-label 21-day MADIAB (Macrobiotic Diet on the improvement of metabolic control in patients with type 2 diABetes and metabolic syndrome) trial was conducted in patients with T2DM comparing the Ma-Pi (Mario Pianesi) 2 diet (C:F:P = 73%:15.2%:11.8%, with 29 g/1000 kcal fiber) with the standard (control) diet (C:F:P = 49.3%:32.3%:18.4%, with 20.5 g/1000 kcal fiber) [36]. Daily average energy intake was 1803 kcal in the Ma-Pi 2 group and 1798 kcal for the control group (*p* = 0.860). The Ma-Pi 2 diet is high in dietary fiber (>50 g/day); it contains a large proportion of complex carbohydrates, whole grains, vegetables and legumes, fermented products, sea salt and green tea, without fat or protein from animal sources and no added sugars [78]. After adjustment for age, sex, BMI and physical activity, there was a significantly greater reduction in FPG (95% CI: 1.79–13.46) and postprandial blood glucose (95% CI: 5.39–31.44) in those patients receiving the Ma-Pi 2 diet (HC-high fiber) than in those with standard diet. Significantly greater reductions in the HbA1c (95% CI: 1.28–5.46), insulin resistance, total cholesterol, LDL-C, LDL-C/HDL-C ratio, BMI and body weight were also found in the Ma-Pi 2 diet group (HC-high fiber) than in the control group. Furthermore, all subjects in the Ma-Pi 2 diet group achieved FPG and postprandial blood glucose target levels (<110 mg/dL for FPG and <140 mg/dL for postprandial blood glucose) at the end of the 21-day dietary treatment.

An RCT by Bozzetto L et al. evaluated the effects of a diet rich in either carbohydrate/fiber with a low GI (moderate carbohydrate/fiber diet; C:F:P = 52%:30% (MUFA 16%):18%, 28 g/1000 kcal for fiber, GI (bread reference) of 48 and mean GL of 15,651) or rich in monounsaturated fatty acids (MUFA diet; C:F:P = 40%:42% (MUFA 28%):18%, 10 g/1000 kcal for fiber, GI (bread reference) of 60 and mean GL of 20,326) on postprandial dyslipidemia in 38 patients with T2DM [37]. After eight weeks, postprandial triglyceride levels decreased after a moderate carbohydrate diet rich in fiber, but increased after MUFA diet (*p* < 0.05). In a meta-analysis of 15 randomized trials involving patients with T2DM, fiber intervention was more effective than placebo on FPG and HbA1c, with an overall reduction in FPG levels of 15.3 mg/dL (95% CI, 8.29–22.52) and in HbA1c levels of 0.26% (95% CI, 0.02–0.51) [79]. The studies in this meta-analysis used a variety of grams of fiber per day in their interventions, ranging from 4–40 g/day of additional fiber, with a mean increase in fiber for the intervention of 18.3 g/day. The most commonly-used fiber intervention dose was a 15-g/day supplement to the usual diet. In another meta-analysis, the effect of dietary fiber on glycemic control of patients with T2DM was evaluated through an analysis of 11 pooled RCT (13 comparisons) of at least and eight-week duration [80]. In studies that evaluated the effect of dietary fiber, the intervention diet was defined as the diet with the highest total fiber content, and the control diet was the diet with the lowest fiber content. In studies that evaluated the effect of a fiber supplement, the intervention was defined as the usual diet plus a soluble fiber supplement, and the control diet was defined as the usual diet or the usual diet plus a placebo or another type of fiber. Diets with foods rich in fiber or fiber supplements caused an absolute reduction of 0.55% (95% CI, −0.96–−0.13) in HbA1c (corresponding to an average reduction of 4.75% (95% CI, −9.35–−0.15)). The observed reduction in HbA1c was achieved with dietary fiber intakes ranging from 37.4–42.6 g/day (considering a 2000 kcal/day diet) or with 3.5–15 g/day of fiber supplements. In addition, pooled data showed a mean FPG reduction of −9.97 mg/dL (95% CI, −18.16–−1.78).

Another positive aspect of HC, high-fiber diet is good acceptability. A higher fiber diet boosts satiety and has the advantage of simplicity because there are no requirements to limit portions [81,82].

## 5. Suggestive Mechanism of the Effects by a High-Carbohydrate Diet on Metabolic Parameters

Fat has a high energy density with more than twice the number of calories per gram compared to carbohydrate; high-fat foods are highly palatable and seem to induce a relatively weak sense of satiety [83]. It has been suggested that dietary fat promotes passive overconsumption, referred to as ‘high-fat hyperphasia’. High carbohydrate foods are usually, but not always, less energy dense than high fat foods and contain dietary fiber, which limits rates of ingestion and digestion, both of which can have a limiting effect on energy intake [84].

A meta-analysis of 37 intervention studies showed that there was a 0.28 kg decrease in body weight every 1% decrease in energy as total fat [85]. Another systematic review found that each 1% decrease in energy from total fat was associated with 0.37 kg (95% CI, 0.15–0.6 kg/%) reduction in body weight [86]. Usually lower fat intake and almost universally higher carbohydrate intakes seem to be in the low fat arms than in the usual fat arms. Energy intake was lower in the low fat group than in the control or usual fat groups, and subgroups suggested that a greater degree of energy reduction in the low fat group was related to greater weight loss.

In fact, studies on subjects in an isocaloric state found that the most prominent weight loss effect of an LC diet occurred during the early stages of the study period, decreasing at the end of the study and finally showing similar changes compared with an HC diet. Furthermore, there is much evidence showing that an HC diet may improve glucose metabolism and weight loss in the case of a high fiber diet, and the possible mechanisms were mentioned above.

The link between diet and plasma lipids and lipoproteins has been recognized since the 1950s. Some studies examining the effect of increased carbohydrate intakes, particularly of sugars, have resulted in elevated triglycerides and sometimes lower HDL-C concentrations and changes in LDL-C composition [87,88,89].

In earlier studies, lower-fat, higher-carbohydrate diets usually contained higher amounts of simple carbohydrate and no additional fiber. Recent work has suggested that simple sugars with high-GI and/or GL might be responsible for the increases in triglycerides [90,91]. Previous studies revealed that increasing carbohydrate intake impaired very low density lipoprotein clearance and reduced whole-body fatty acid (FA) oxidation [92,93]. HC feeding causes a shift in the intracellular partitioning of FAs toward esterification and away from oxidation. For example, elevated insulin concentrations on an HC diet may stimulate the pathway of fatty synthesis, raising the malonyl-CoA concentrations, which would divert FAs from oxidation into triacylglycerol synthesis. This may explain increased triacylglycerol production by the liver, but may also affect FA oxidation by muscle [94,95]. On the contrary, HC diets with high fiber have shown decreased cholesterol and triglyceride levels. The likely mechanisms explaining the beneficial effects of high fiber on lipid profiles have been suggested. Soluble fibers have been shown to increase the rate of bile excretion and intestinal viscosity, so reducing serum total cholesterol and LDL-C [72]. Specific short chain fatty acid production via fermentation has been shown to inhibit cholesterol synthesis [96]. Regarding LDL-C, the lowest intake of total fat was associated with a trend of decrease in LDL-C. A greater reduction in saturated or trans-fat was associated with a greater decrease in LDL-C. the Women’s Health Initiative (WHI) trial was not designed to assess strategies for increasing carbohydrate intake; however, increases in carbohydrate were associated with a decrease in energy from total fat, and the study provides long-term data on the stability of lipoprotein risk during a higher carbohydrate diet for a long time over six years [97]. Furthermore, we need to consider possible explanations of studies not supporting the benefits of HC diets. It has been postulated that one reason for the apparent failure of HC diets is that they may provoke physiological adaptations—slowing metabolic rate and increasing hunger—that antagonize ongoing weight loss [98]. Preliminary studies suggest that the reduced insulin secretion associated with LC and low-GI diets may attenuate these adaptations, facilitating long-term weight-loss maintenance and reducing hyperinsulinemia (the carbohydrate-insulin model) [93]. The conflicting results of studies on HC diets are largely because of the fact that it was not recognized that carbohydrates are a heterogeneous class of nutrients [7,15].

## 6. Conclusions

Numerous studies have compared the effectiveness of HC diets with LC diets on weight reduction, glucose control and lipid profiles in patients with T2DM and have shown conflicting results. Moreover, over recent years, the debate on the pros and cons of HC diets has intensified, due to potential untoward effects of carbohydrates on postprandial glycemic response and plasma lipid levels, such as an increase in triglyceride and a decrease in HDL-C levels (Table 3). However, recent data suggest that an HC diet causes no deterioration in glycemic control and lipid profiles, specifically triglyceride and HDL-C. Furthermore, an HC diet has similar effects on an LC diet, if composed with high-fiber, low-GI/low-GL food, rather than refined carbohydrate or high-GI food. At the same time, these studies highlight the difficulties in achieving good compliance with dietary treatment in the long term, independent of the macronutrient composition of the diet. Choosing an easy diet to follow is important in MNT for T2DM.

There are several limitations to studies regarding an HC diet. The lack of a standardized definition for an HC diet makes it difficult to interpret the effects of dietary carbohydrate. Furthermore, the studies have been mainly derived from Western populations (Table 4). Data derived from large RCTs involving populations of diverse ethnicity, with a traditional HC diet, are required. In view of the results available to date, it is difficult to define the ideal amount of carbohydrate, and it may be necessary to determine an individualized guide for carbohydrate intake, considering GI/GL and dietary fiber contents.

## Figures and Tables

**Table 1:** Suggested definitions of high carbohydrate.

Amount of Carbohydrate	Reference
High carbohydrate: >65% of total energy	Liebman [4]
Typical carbohydrate diets: 45%–65%	
Moderately-restricted carbohydrate diet: 26%–44%	
Low carbohydrate: <130 g/day (which represents 26% of calories of a 2000 calorie diet)	
Very low carbohydrate diet: 20–50 g of carbohydrate or 5%–15% of total energy	
High carbohydrate: >65%	Naude et al. [11]
Balanced carbohydrate: 45%–65%	(based on recommendation range from USA, Canada, Australia, New Zealand and Europe)
Low carbohydrate: <45%	
High carbohydrate: >45% of total energy	Feinman et al. [10], Accurso et al. [12]
Moderate carbohydrate: 26%–45%	(based on recommendation target on ADA websites, on the 2010 dietary guidelines for Americans and carbohydrate consumption (NHANES)
Low carbohydrate: <130 g/day or <26%	
Very low carbohydrate ketogenic: 20–50 g/day or 10% of the 2000 kcal/day diet	
By carbohydrate/fat ratio;	Kodama et al. [14]
High carbohydrate: 1.67–7.3	
Low carbohydrate: 0.6–1.56	

ADA: American Diabetes Association; NHANES: National Health and Nutrition Examination Survey.

**Table 2:** Medical nutrition therapy recommendations for subjects with T2DM from various organizations: carbohydrate focusing comparison.

	ADA (2016)	KDA (2015)	JDA (2013)	CDA (2013)	EASD (2004)
**Macronutrient distribution**	Use of individualized assessment because evidence suggests no one ideal distribution for all people	The intake of carbohydrate, protein and fat should be individualized according to the patient’s eating patterns, preferences and metabolic goals	50%–60% of carbohydrate, 1.0–1.2g/kg/ideal body weight, rest of the energy for fat (no consensus as to the lower normal limits for carbohydrate, desirable not more than 60%)	Individualization within the following ranges: 45%–60% carbohydrate, 15%–20% protein, 20%–35% fat of total energy	Ranges of 45%–60% carbohydrate, 10%–20% protein, <35% fat of energy
**Glycemic index and Glycemic load**	Substituting low glycemic load foods for higher glycemic load foods may be beneficial	50%–60%	50%–60%	45%–60% choose food sources from a low glycemic index	low glycemic index foods are suitable as carbohydrate-rich choices
**Fiber and whole grain**	Consume at least the amount recommended for the general public (14 g/1000 kcal or 25 g/day for women and 38 g/day for men)	Including various sources such as whole grains, should be 20–25 g/day (12 g/1000 kcal/day)	Fruit is limited to up to 1 unit (<80 kcal/day)	Consume higher intake than those for the general public (25–50 g/day or 15–25 g/1000 kcal)	Consume fiber intake >40 g/day (or 20 g/1000 kcal/day) with half as soluble; choose cereal-based foods high in fiber and whole grains
**Sucrose and fructose**	Limit or avoid intake of sugar-sweetened beverages		Intake of sweets, jams or soft drinks must be minimized	Added sucrose or fructose can be substituted for other carbohydrate as a mixed meal up to a maximum of 10% total daily energy intake	Moderate intake of free sugars (up to 50 g/day) recommended without exceeding 10% total energy

References: American Diabetes Association (ADA) 2016 [15], Korean Diabetes Association (KDA) 2015 [20], Japan Diabetes Association (JDA) 2013 [19], Canadian Diabetes Association (CDA) 2013 [16], European Association for the Study of Diabetes (EASD) 2004 [17].

**Table 3:** Positive and negative attributes of high-carbohydrate diet.

		Solution for Cons
Pros	Considerable body weight reduction	
	Efficient fasting glucose control/HbA1c	
	Decrease in LDL-C	
Cons	Raise in TG	HC with low GI/high fiber counter these lipid abnormalities
	Decrease in HDL-C	
	Increase postprandial glucose/insulin response	HC with low GI/high fiber counter these lipid abnormalities

**Table 4:** Summary of trials. HC, high carbohydrate; LF, low fat; VLC, very low carbohydrate; GI, glycemic index; GL, glycemic load.

Study	Country	Participants	*n*	Duration	Compared Diets	Amount of Macronutrients (C:F:P, % of Total Energy Intake)	GI/GL/Fiber	Main Source of Carbohydrate
McDougall et al. [21]	USA	14.4% among participants had DM	1615	7 days	No comparison group (HC/LF, starch-based diet only)	81%:7%:12%	Not presented	Common starches, including wheat flour products, corn, rice, oats, barley, quinoa, potatoes, sweat potatoes, beans, lentils
Guldbrand et al. [22]	Sweden	Adults with T2DM	61	2 years	HC(LF) diet vs. VLC diet	HC(LF):55%–60%:30% (<10% saturated fat): 10%–15% VLC: 20%:50%:30%	Not presented	
Brehm et al. [23]	USA	Overweight/obese with T2DM	124	1 year	HC diet vs. high MUFA diet	HC: 60%:25%:15% High-MUFA: 45%:40% (20% MUFA):15% Both diet: calorie restriction (200–300 kcal/day)	Not presented	
Meher et al. [24]	India	Newly diagnosed T2DM, treatment-naive	48	Postmeal 0, 2, 4 h	No comparison group (HC diet only)	HC (mixed meal breakfast): 79%:13%:8%	Not presented	Biscuits and sweetened milk
Krebs et al. [27]	New Zealand	T2DM	419	1 year and following 1 year	HC vs. High-Protein, Low-Fat(HPLF) diet	HC: 55%:30%:15% HPLF: 40%:30%:30% Both diet: calorie restriction (500 kcal/day)	Fiber: 1 year in study (HC vs. HPLF = 23.8 g vs. 25 g/day) Following 1 year after study (HC vs. HPLF = 23.7 g vs. 23.2 g/day)	
Larsen et al. [28]	Australia	Overweight/obese with T2DM	99	1 year	HC vs. HP diet	HC: 55%:30%:15% HP: 40%:30%:30% (3-month energy restrictive period (30%) followed by 9 months)	Both diets were recommended low GI diet	
Hsu et al. [31]	USA	Adults at risk for DM (Caucasian Americans and East Asian Americans; Korean, Chinese and Japanese)	50	16 weeks	Control group (16 weeks of Traditional Asian Diet (TAD)) vs. intervention group (8 weeks of TAD, followed by 8 weeks of Traditional Western Diet (TWD))	TAD (HC): 70%:15%:15% TWD: 50%:34%:16%	TAD (HC): 15 g fiber/1000 kcal TWD: 6 g fiber/1000 kcal	
Sacks et al. [54]	USA	Overweight/obese adults	163	5 weeks	High GI/HC vs. Low GI/HC vs. High GI/VLC vs. Low GI/VLC diet	HC: 58% carbohydrate, VLC: 40% carbohydrate High GI: ≥65 on the glucose scale Low GI: ≤45 on the glucose scale (each diet was based on DASH-type diet)	Fiber: - High GI: averaged 31 g - Low GI: averaged 35 g	
Wolever et al. [52]	Canada	T2DM managed by diet only	162	1 year	High GI vs. low GI vs. VLC/High-MUFA	High GI: 47%:31%:22% low GI: 52%:27%:21% VLC/high-MUFA: 39%:40%:21%	High GI: GI 63 Low GI: GI 55 VLC/high-MUFA: GI 59	High GI/low GI diet: starch carbohydrates
Farvid et al. [63]	Iran	Adults with T2DM	640	Cross-sectional study	According to the Quartile of GI/GL		Quartile of GI: <54.1, 54.1–58.7, 58.8–63.5, >63.5 Quartile of GL : <129.6, 129.6–171.2, 171.3–212, >212	
